# Covariate-Adjusted Response-Adaptive Randomization for Multi-Arm Clinical Trials Using a Modified Forward Looking Gittins Index Rule

**DOI:** 10.1111/biom.12738

**Published:** 2017-07-06

**Authors:** Sofía S. Villar, William F. Rosenberger

**Affiliations:** 1 MRC Biostatistics Unit, School of Clinical Medicine, University of Cambridge, Cambridge, U.K.; 2 Department of Statistics, George Mason University, U.S.A.

**Keywords:** Adaptive designs, CARA randomization, Ethics, Multi-armed bandit, Sequential allocation

## Abstract

We introduce a non-myopic, covariate-adjusted response adaptive (CARA) allocation design for multi-armed clinical trials. The allocation scheme is a computationally tractable procedure based on the Gittins index solution to the classic multi-armed bandit problem and extends the procedure recently proposed in Villar et al. (2015). Our proposed CARA randomization procedure is defined by reformulating the bandit problem with covariates into a classic bandit problem in which there are multiple combination arms, considering every arm per each covariate category as a distinct treatment arm. We then apply a heuristically modified Gittins index rule to solve the problem and define allocation probabilities from the resulting solution. We report the efficiency, balance, and ethical performance of our approach compared to existing CARA methods using a recently published clinical trial as motivation. The net savings in terms of expected number of treatment failures is considerably larger and probably enough to make this design attractive for certain studies where known covariates are expected to be important, stratification is not desired, treatment failures have a high ethical cost, and the disease under study is rare. In a two-armed context, this patient benefit advantage comes at the expense of increased variability in the allocation proportions and a reduction in statistical power. However, in a multi-armed context, simple modifications of the proposed CARA rule can be incorporated so that an ethical advantage can be offered without sacrificing power in comparison with balanced designs.

## 1 Introduction

The Gittins index rule (Gittins and Jones, 1979) was developed as an optimal solution to the classic multi-armed bandit problem. In the context of a clinical trial to test the effectiveness of several treatments with an infinite number of patients, it also provides a deterministic patient allocation rule that aims to optimize patient benefit on average. In order to do so, the rule must dynamically address the ethical conflict between learning (efficiency/power) and earning (patient benefit/ethics) after every patient is treated, its outcome observed and considering the potential outcomes of the future patients, given the observed history.

The multi-armed bandit problem and the Gittins index are based on a set of assumptions which may be restrictive when considered from a practical point of view (Villar et al., 2015). Particularly important assumptions include the infinite size of the trial, the observability of each patient’s outcome before treating the next patient, and the lack of randomization of the resulting patient allocation rule. Any extensions of the original model that result from relaxing some (or all) of these assumptions would, in general, require either finding an appropriate extension of the Gittins index rule for the relaxed model (e.g., an index for the finite horizon problem investigated by Villar et al. (2015)), or otherwise relying on a computational solution using dynamic programming (e.g., as in Cheng and Berry (2007) or Williamson et al. (2017)). The latter approach requires the problem to be of a tractable size. An alternative approach was proposed in Villar et al. (2015) where the Gittins index rule was used to define a non-myopic response-adaptive randomized procedure for the design of finite-sized trials—namely, the block randomization procedure referred to as the *forward looking Gittins index* (FLGI).

Incorporating covariates into the multi-armed bandit model is one such extension. There are at least two reasons why this would be relevant. First, including covariate information into the model would imply relaxing the assumption that observations of a given treatment are exchangeable (i.e., that subjects receiving the same treatment arm have the same probability of success). This would, in turn, allow for the inclusion of treatment–covariate interactions and the modified bandit model with covariates would maximize patient benefit by assigning more patients to a superior treatment, given their covariate profile. Second, methods that promote balance on important known covariates have become a general standard among practicing clinical trialists. However, there are many relevant instances in which balance does not lead to efficiency or ethically attractive designs, as shown in Rosenberger and Sverdlov (2008). A bandit model with covariates would illustrate this conflict, as balance on covariates would never be achieved by its optimal solution rule if treatments are perceived differently among covariate groups.

In this article, we address the issue of introducing covariates into the classic multi-armed bandit model. We first present a deterministic implementation of the Gittins index that makes use of covariate information, and then use it to define a covariate-adjusted response-adaptive (CARA) randomization procedure that is non-myopic and applied to blocks of patients rather than individuals, as suggested by Rosenberger and Lachin (1993). The resulting CARA procedure sacrifices a small amount of expected patient benefit in order to introduce randomization. Our procedure differs from existing CARA procedures by optimizing patient benefit in an unconstrained fashion, rather than with a constraint to preserve power (e.g., Rosenberger, Vidyashankar, and Agarwal, 2001).

The bandit literature has very few relevant papers following the lines of Gittins’ work. Woodroofe (1979) studied the optimal policy structure of a simplified special case of a one-armed bandit problem with a covariate. Clayton (1989) concludes that the existence of the index function depends on the functional form of the assumed relation between outcome and covariate (or *link* function), among other assumptions. Yet, for the usual logit function, the existence of the index is only conjectured under certain constrains on the parameter space. More recently, there has been work on randomized bandits with covariates by Yang and Zhu (2002). The authors introduce a myopic randomized solution that is asymptotically consistent. We take a different approach, concentrating on the introduction of covariates and randomization to the Gittins index, by taking into account future sequences of allocations and covariate values under the Gittins rule. Our simple, heuristic approach thereby aims to achieve a near-optimal mean total rewards criterion in a computationally feasible way.

In Section 2, we introduce the modified Gittins’ rule and demonstrate its application in a clinical trial setting with a binary outcome and a binary covariate. We introduce our probabilistic implementation of the modified Gittins index, which we call the covariate-adjusted response-adaptive forward looking Gittins index (CARA FLGI), in Section 3. In Section 4, we compare our approach to alternative procedures by performing simulations, including scenarios in the context of a recently published trial (Schortgen et al., 2012). We briefly discuss extensions to multiple polychotomous covariates in Section 5. We draw conclusions in Section 6.

## 2 The Gittins Rule for a Model with Covariate Information

Consider a clinical trial to test the effectiveness of *T* experimental treatments against a control treatment on a fixed sample of *N* patients. Assume *T* and *N* are fixed and known. Before a patient *n* (*n* = 1, … , *N*) is allocated to treatment *t* (*t* = 0, … , *T*), where *t* = 0 denotes the control, a binary characteristic or covariate *Z_n_* is observed for patient *n*. Immediately after making a treatment decision, that is, patient *n* receives treatment *t*, a binary outcome variable (sucess/failure) *Y*_*t*,*n*_ is observed with Pr(*Y_t,n_* = 1) = *p_t_*, the true unknown response probability for treatment *t*. Assume that *Y*_*t*,*n*_ = 1 if the treatment is successful and 0 otherwise. Patients enter the trial one-by-one and the outcome for patient *n* is observed before patient *n* + 1 appears.

Let *Z_n_* ~ Bernoulli(*q*) for all *n* = 1, … , *N*, where *Z_n_* = 0, 1, respectively, indicates patient *n* is covariate negative or covariate positive. Let *Y*_*t*,*n*_ ~ Bernoulli (*p_t_*(*z_n_*)) for all *n* = 1, … , *N*. For example, we can assume that for all *n*: *p_t_* (*z_n_*) = *Expit* (*α_t_* + *β_t_z_n_*) for *t* = 0, … , *T* with *α_t_* and *β_t_* being unknown parameters and where we have defined

$$Expit(u)=\frac{exp(u)}{1+exp(u)},\;Logit(u)=log\left(\frac{u}{1-u}\right).$$

We will further assume that the probability of the covariate taking value 1, that is, *q*, is known. The goal is to find a patient allocation procedure that, taking into account the covariate and outcome observations available at each time, maximizes the expected total number of successes after the *N* patients have been treated. The resulting optimization problem will no longer admit a Gittins index solution as each arm’s state variable includes the covariate data, which continues to evolve regardless of whether a treatment is allocated or not, making the bandit formulation *restless*.

In order to define a Gittins index heuristic solution for this problem with covariates, we will reformulate it as follows. We will consider that for every treatment–covariate combination there exists a independent arm, which will be indexed by its covariate value and treatment arm label, that is, *zt*. For example, the arm 00 is the arm corresponding to covariate value 0 and the control treatment. Therefore, there will be 2 (*T* + 1) arms in this reformulated version of the *classic* multi-armed bandit problem, each of which has a success rate *p_zt_*.

Let each parameter *p_zt_* be assigned a Beta(*s*_*zt*,0_, *f*_*zt*,0_) prior density at the start of the trial, where *s*_*zt*,0_ and *f*_*zt*,0_ denote prior beliefs about the relative chances of success and failure of arm *zt,* respectively. Given the conjugacy of the prior and Bernoulli distributed outcome, these priors are converted into beta posteriors for *p_zt_* via Bayes theorem as patients enter the trial, are assigned to a treatment arm given their observed covariate *z_n_* and subsequently experience a success or a failure. Let **X**_*zt*,*n*_ = (*s_zt_*_,0_ + *S*_*zt*,_*_n_*, *f_zt_*_,0_ + *F*_*zt*,*n*_) be the 2 state-vector of available information on arm *zt* before treating patient *n,* where the random vector (*S_zt_*_,*n*_, *F_zt_*_,*n*_) represents the number of successful and unsuccessful outcomes for arm *zt* up to patient *n*. The posterior for *p_zt_* after having treated patient *n* and observing *s*_*zt*,*n*_ successes and *f_zt_*_,*n*_ failures, is: *f* (*p*_*zt*_|x*_zt_*_,_*_n_*) ~ Beta(*s_zt_*_,0_ + *s_zt_*_,_*_n_*, *f_zt_*_,0_ + *f_zt_*_,*n*_) with its posterior mean being $\text{E}\left[p_{zt}|{\text{x}}_{zt,n}\right]=\frac{s_{zt,n}+s_{zt,0}}{s_{zt,n}+f_{zt,n}+s_{zt,0}+f_{zt,0}}.$ Finally, let *a*_*zt*,*n*_ be the binary indicator variable denoting whether patient *n* + 1 is assigned to arm *zt* or not. The multi-armed bandit optimization problem is to find an allocation rule *π* such that:

(1) $$V_{D}^{*}({\tilde{\text{x}}}_{0})=\underset{\pi \in \Pi }{\text{max}}\;{\text{E}}^{\pi }\left[\left(\sum_{n=0}^{N-1}\sum_{t^{\prime }=1}^{2(T+1)}d^{n}\text{E}[p_{t^{\prime }}|{\text{x}}_{t^{\prime },n}]a_{t^{\prime },n}\right)|{\tilde{\text{x}}}_{0},\right].$$

where *t*′ = {1, 2, … , *T*, *T* + 1, *T* + 2, … 2(*T* + 1)} stand for 00, 01, … , 0*T*, 10, 11, … , 1*T*, respectively, ${\tilde{\text{x}}}_{0}={\left({\text{x}}_{t^{\prime },0}\right)}_{t^{\prime }=1}^{2(T+1)}$ is the initial joint state with all the prior parameters, **Ε**^*π*^[·] denotes expectation under allocation rule ***π***, and *d* is a discount factor (i.e., 0 ≤ *d* < 1) introduced for reasons of tractability, so that a trial of infinite size (*N* = ∞) can be assumed. Thus, $V_{D}^{*}({\tilde{\text{x}}}_{0})$ is the optimal expected total discounted value function conditional x̃_0_ over П, the family of admissible allocation rules, which for this particular reformulated model are those for which for all *z_n_* = 0 it holds that $\sum_{t^{\prime }=1}^{T+1}a_{t^{\prime },n}=1\;\text{and}\;\sum_{t^{\prime }=T+2}^{2(T+1)}a_{t^{\prime },n}=0$ while for all *z_n_* = 1 it holds that $\sum_{t^{\prime }=1}^{T+1}a_{t^{\prime },n}=0\;\text{and}\;\sum_{t^{\prime }=T+2}^{2(T+1)}a_{t^{\prime },n}=1$ (in words, those rules that allocate an arm *zt* that is available for covariate value *z_n_*). Put simply, (1) is the maximum average (discounted) number of patients responses attainable given the initial information on the available treatments and covariates before the start of the trial.

The solution to (1) could in principle be found via dynamic programming, using a backward induction algorithm. However, this becomes computationally infeasible for relatively small values of *N* and 2(*T* + 1) and is further extremely difficult to implement in practice. A computationally tractable solution to (1) when considered for *N* = ∞ based on the indices proposed by Gittins and Jones (1979) would be to allocate patient *n* with covariate *z_n_* to the arm that is available for covariate *z_n_* with the highest Gittins index at time *n* – 1. For arm *zt* at time *n*, and stopping time *τ*, this is denoted $𝒢({x_{\mathrm{zt}},}_{n})$ where:

(2) $$𝒢({\text{x}}_{zt,n})=\underset{\tau \geq 1}{\sup}\frac{\text{E}\left[\left(\sum_{i=0}^{\tau -1}\text{E}\left[p_{zt}|{\text{x}}_{zt,n+i}\right]d^{i}\right)|{\text{x}}_{zt,n}\right]}{\text{E}\left[\left(\sum_{i=0}^{\tau -1}d^{i}\right)|{\text{x}}_{zt,n}\right]}.$$

Here (2) represents the ratio between the total expected discounted number of successes observed after allocating arm *zt* from patient 1 up to *τ* given the initial information on arm *zt* and the total expected discounted number of patients treated with arm *zt* from patient 1 to *τ* given the initial information on arm *zt.* We will refer to this solution as the CARA Gittins index rule (CARA GI). Notice that the index defined by (2) depends only on the current information state of arm *zt.* These Gittins indices can be calculated by solving the problem of allocating patients optimally between treatment *zt* and a known treatment which yields a constant reward. For a detailed explanation of how the Gittins index rule is deployed and a table with values see Tables 4 and 5 of Web Appendix A in Villar et al. (2015).

## 3 The Covariate-Adjusted Response-Adaptive Forward-Looking Gittins Index Rule

Following the approach introduced in Villar et al. (2015), we will assume that instead of enrolling the *N* patients one-by-one, patients are enrolled in groups of size *b* over *J* stages, so that *J* × *b* = *N*. We wish to specify a CARA rule based on the CARA Gittins index rule that sequentially randomizes the next *b* patients among the *T* + 1 treatments at stage *j* (*j*=1,… , *J*) given the data up to block *j* – 1 and each of the patients observed covariate value *z_n_*. This translates to determining *π*_*zt*,*j*_, where: *π*_*zt*,*j*_ = the probability of allocation for patients to treatment *zt* at stage *j* (*j* = 1,… , *J*), which is common to all patients with covariate value *z* in block *j*, when using the CARA Gittins index rule as defined in Section 2 and given data observed up to the stage *j* – 1 (and thus patient (*j* – 1) × *b*), denoted by ***x̃***_(*j*–1)*b*_. Note that ***x̃***_(*j*–1)*b*_ can be written as a 2(*T* + 1) × 2 matrix in which row *t*′ represents the parameters of arm *t*′’s current posterior distribution up to patient (*j* – 1)*b*. This marginal probability is obtained via the procedure next explained. Let *b̄*_*Z*=1_ represent the expected number of patients with covariate positive value in a block of size *b*, that is, *b̄*_*Z*=1_ = *b* × *q* and let $a_{zt,n}^{GI}$ be the binary variable representing if arm *zt* is allocated to patient *n* under the CARA Gittins index rule $\left(a_{zt,n}^{GI}=1\right)$ or not $(a_{zt,n}^{GI}=0).$ The formula below represents the probability of allocating each combination arm *zt* in a block of size *b̄*_*Z*=*z*_ given that the first patient in the block has an observed covariate value of *Z_n_* = *z* and when using the Gittins index rule:

(3) $$\pi_{zt,j}(Z_{n}=z)=\frac{1}{{\bar{b}}_{Z=z}}\sum_{n=(j-1)b+1}^{jb}\left[\sum_{{\tilde{x}}_{n-1}\in \Omega_{n-1}}Pr(a_{zt,n}^{GI}=1|{\tilde{X}}_{n-1}={\tilde{x}}_{n-1})Pr({\tilde{X}}_{n-1}={\tilde{x}}_{n-1}|{\tilde{X}}_{(j-1)b}={\tilde{x}}_{(j-1)b})\right]$$

Here Ω_*n*–1_ represents the set of all possible values for ***X̃***_*n*–1_ given initial data ***x̃***_(*j*–1)*b*_ for every future patient *n* in (*j* – 1)*b* + 1, … , *jb* under the CARA Gittins index rule described in Section 2 (summarized by $a_{zt,n}^{GI}$). Each term of the summation within the square brackets of (3) represents the joint probability of allocating a future patient *n* with covariate *z* in block *j* to treatment *t* and the current information state at patient *n* – 1, given the data at the beginning of block *j* – 1 and the probability of a patient being covariate positive *q*.

In order to compute the CARA FLGI allocation probabilities for block *j* and arm *zt* (which do not depend on the covariate value of the first patient in the block), we average the probabilities calculated in (3) as follows:

(4) $$\pi_{zt,j}=\sum_{Z_{n}=\{0,1\}}\pi_{zt,j}(Z_{n}=z)Pr(Z_{n}=z).$$

### 3.1 Worked Example

To illustrate how these probabilities are computed, implemented and their computational cost, we present the simplest case of a trial testing a control treatment (*t*=0) against an experimental treatment (*t*=1) with a block of size 2 (i.e., *b* = 2 and 2(*T* + 1) = 4) and a binary covariate *Z* = {0, 1}. Suppose further that in the first block of the trial, having started with Beta(1,1) priors for all the *t*′ = 4 arms, the resulting allocation was: one covariate negative patient to control (i.e., arm *t*_00_)—one success—and a covariate positive patient to experimental *t*_11_—another success—and no patients allocated to the remaining arms (i.e., *t*_01_, *t*_10_). Hence, for the second block the priors for each combination arm are ***x̃***_2_ = [(2, 1); (1, 1); (1, 1); (2; 1)].

**Figure 1:**
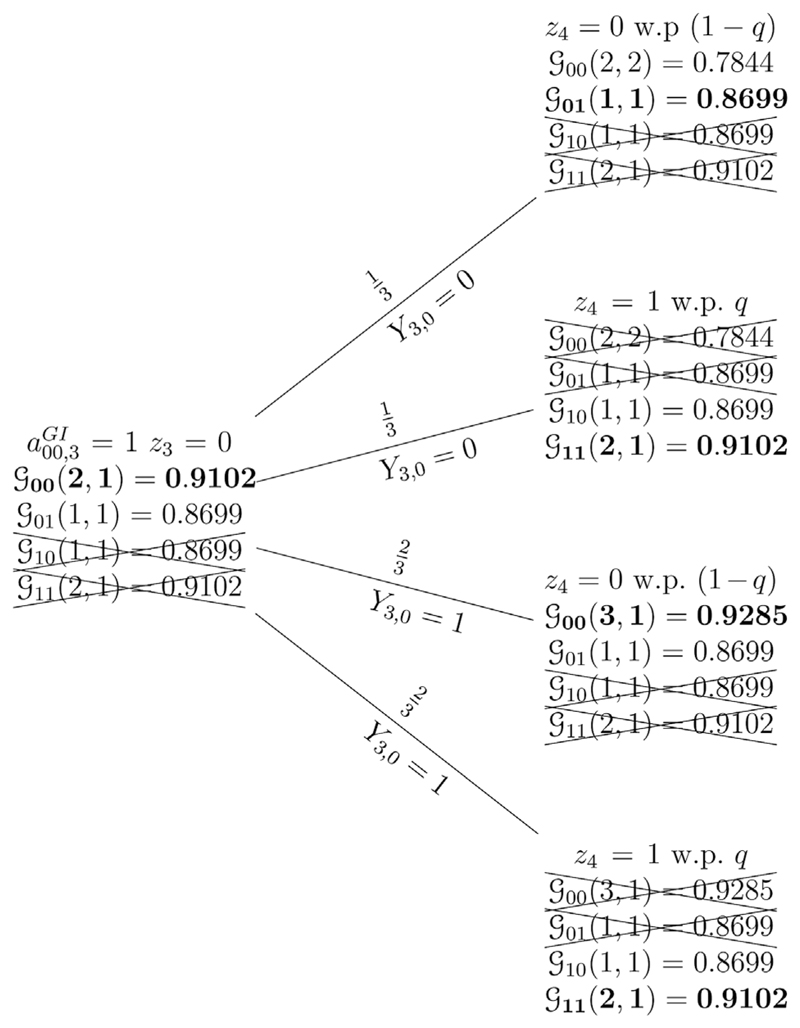
The CARA FLGI rule and a probability tree of all trial histories using the Gittins index rule when *K =* 2, *b =* 2, *Z* = {0, 1} w.p {(1 – *q*), *q*}, and ***x̃***_2_ = [(2, 1); (1, 1); (1, 1); (2, 1)]. Bold text indicates the allocated combination arm under the Gittins index rule $\{a_{zk,t}^{GI}\}.$ Gittins index values used correspond to *d* = 0.99. Gittins indices that have been crossed out indicate arms that are not available for a patient given her covariate value.

Figure 1 shows, via a probability tree, how the CARA FLGI probabilities for block 2 given the data in block 1 are computed for the first patient in the block being covariate negative value. Given that the control treatment has the maximum Gittins index, the allocation for the first patient of the second block who has a covariate negative value (i.e., patient 3 with *z*_3_ = 0) is deterministic. It follows that $𝒫r(a_{00,3}^{GI}=1|{\tilde{\text{x}}}_{2})=1\;\text{and}\;𝒫r(a_{01,3}^{GI}=1|{\tilde{\text{x}}}_{2})=0.$

When the second patient of the second block is to be allocated (i.e., patient 4), given that we have allocated the control treatment to the first patient, two possible outcomes can occur. If a success occurs, which happens with probability 2/3, then the control treatment is allocated again if the next patient is covariate negative. If a failure occurs and the next patient is covariate negative then the experimental treatment is allocated. If the next patient is covariate positive, the experimental treatment is allocated regardless of the previous outcome. Hence, $𝒫r(a_{00,4}^{GI}=1|{\tilde{\text{x}}}_{2})=\sum_{{\tilde{x}}_{3}\in \Omega_{3}}Pr(a_{00,4}^{GI}=1|{\tilde{\text{X}}}_{3}={\tilde{x}}_{3})𝒫r({\tilde{\text{X}}}_{3}={\tilde{x}}_{3}|{\tilde{\text{x}}}_{2})$ reduces to 2/3(1 – *q*), while $𝒫r(a_{01,4}^{GI}=1|{\tilde{\text{x}}}_{2})=\sum_{{\tilde{x}}_{3}\in \Omega_{3}}Pr(a_{01,4}^{GI}=1|{\tilde{\text{X}}}_{3}={\tilde{x}}_{3})𝒫r({\tilde{\text{X}}}_{3}={\tilde{x}}_{3}|{\tilde{\text{x}}}_{2})$ reduces to 1/3(1 – *q*). Using (3), we can obtain *π*_00,__2_(*Z*_3_ = 0) = (1 + 2/3(1 – *q*))/1 and *π*_01,2_(*Z*_3_ = 0) = (0 + 1/3(1 – *q*))/1. A similar reasoning for the first patient in the block being covariate positive, given the data in block 1, yields *π*_00,2_(*Z*_3_ = 1) = (0 + 1/3(1 – *q*) + 2/3(1 – *q*))/1 = (1 – *q*) and *π*_01,2_(*Z*_3_ = 1) = (0 + 0)/1 = 0. Using (4), we can compute that: *π*_00,2_ = (1 + 2/3(1 – *q*)) * (1 – *q*) + (1 – *q*) * *q* and *π*_01,2_ = (1/3(1 – *q*)) * (1 – *q*) + 0 * *q*. If *q* = 1/2, then *π*_00,__2_ = 11/12 and *π*_01,__2_ = 1/12. Analogous calculations result in *π*_10,2_ = 1/12 and *π*_11,2_ = 11/12.

From this example, it is clear that the computational cost of computing the *π*_*zt*,*j*_’s, which depend on the joint state for the 2(*T* + 1)-arms, that is, ***x̃**_n_* (instead of the 1 arm state ***x**_n_*), will grow exponentially as *b* and *T* increase. Hence, we use a Monte-Carlo algorithm for this purpose. It follows that for *Z_n_* = *z* if we plug (3) into (4) the *π*_*zt*,*j*_’s add up to 1.

## 4 Simulation Study

In the following, we assume that responses for treatment *t* satisfy the following logistic model: *Logit* (*p_t_*(*z_n_*)) = *α_t_* + *β_t_z_n_*, where *α_t_* is treatment’s *t* effect, and *β_t_* is the effect due to the binary covariate *Z* in treatment group *t*. The parameter of interest is the covariate-adjusted treatment difference, defined as *α_t_* – *α*_0_ for *t* = 1, … , *T*. The covariate *Z* is assumed to be independently distributed as a Bernoulli(*p_z_*), where *p_z_* is known before the start of the trial. Notice that the model allows for treatment–covariate interaction, since the covariates effects coefficients *β_t_*’s are allowed to vary across treatments.

We now evaluate the properties of the proposed CARA procedures by simulation, focusing on operating characteristics that include measures of validity, efficiency, balance and ethics. The validity of the procedures is measured by the average significance level or type I error rate *α* of the test under the null hypothesis. To assess the chance of a type I error being made under the global null in a multi-arm setting, we report the family-wise error rate *ᾱ*. This is the probability of rejecting at least one true null hypothesis. The Bonferroni method is used to account for multiple testing and ensure that *ᾱ* ≤ *α*, that is, all hypothesis whose p-values are less than $\frac{\alpha }{K}$ are rejected (with *α* = 0.05). The efficiency of procedures is measured by the average statistical power (1 – *β*) of the test used. To assess power in a multi-arm setting, we calculate the probability of rejecting the null for the truly best treatment under each assumed scenario. The balance measures of procedures considered are the average allocation proportion per treatment *N_t_*(*N*)/*N* for *t* = 0, … , *T*. For a measure of (im)balance and ethics, we consider the average allocation proportion of patients within *Z* category assigned to the best treatment for that category *N*_(*zt*)*_ (*N*)/*N*. The ethical performance of the procedures is assessed by the expected total number of treatment failures (ENF) and the average proportion of patients assigned to the best treatment p*, defined as *pz* × *N*_(1*t*)*_ (*N*)/*N* + (1 – *pz*) × *N*_(0*t*)*_ (*N*)/*N*. Finally, for a combined measure (*CM*) of the power-ethics trade-off, we report the percentage of trial realizations in which: (1) 85% or more patients with biomarker 0 received the best arm under *H*_1_ and (2) resulted in a statistically significant treatment difference.

We compare both the deterministic and randomized GI-based CARA procedures to the following established CARA allocation procedures for the logistic regression model:

(1) Equal randomization (ER): patients within a covariate group are allocated between the *T* experimental arms and the control arm with a fixed and equal probability of $\frac{1}{\left(T+1\right)}.$
(2) CARA 1: Rosenberger, Vidyashankar, and Agarwal (2001) propose the allocation target:
  (5) $$\rho_{0}^{1}(z)=\frac{p_{0}(z)/q_{0}(z)}{p_{0}(z)/q_{0}(z)+p_{1}(z)/q_{1}(z)},$$
  where *p_t_*(*z*) = *Expit*(*α_t_* + *β*_*t*_*z*) and *q_t_*(*z*) = 1 – *p_t_*(*z*).
(3) CARA 2: the allocation ratio proposed in Rosenberger et al. (2001):
  (6) $$\rho_{0}^{2}(z)=\frac{\sqrt{p_{0}(z)}}{\sqrt{p_{0}(z)}+\sqrt{p_{1}(z)}},$$
(4) CARA 3: covariate-adjusted version of Neyman allocation:
  (7) $$\rho_{0}^{3}(z)=\frac{\sqrt{p_{1}(z)q_{1}(z)}}{\sqrt{p_{0}(z)q_{0}(z)}+\sqrt{p_{1}(z)q_{1}(z)}},$$
(5) CARA 4: covariate-adjusted version of optimal allocation:
  (8) $$\rho_{0}^{4}(z)=\frac{\sqrt{p_{1}(z)}q_{1}(z)}{\sqrt{p_{0}(z)}q_{0}(z)+\sqrt{p_{1}(z)}q_{1}(z)},$$
(6) Thompson CARA Sampling (TS) (Thompson, 1933): patients within a covariate group are allocated between the experimental arms and the control arm with a probability proportional to the posterior probability that *p_zt_* is the largest response rate given the observed data. Specifically, we defined $\pi_{zt,j}^{TS}=\frac{𝒫r{\left({\text{max}}_{i}\;p_{i}=p_{zt}|{\tilde{\text{x}}}_{(j-1)b}\right)}^{c}}{\sum_{i|Z=z}𝒫r{\left({\text{max}}_{i}\;p_{i}=p_{zt}|{\tilde{\text{x}}}_{(j-1)b}\right)}^{c}}$ with $c=\frac{J}{2T}.$
(7) Stratified permuted block design (SPBD): the stratum of the current patient is determined based on the patient’s observed covariate value. Within that stratum allocations are made using a permuted block of size *m (m* is reported for every simulation).

Note that in choosing which rules to compare our procedure against, we wanted to include all related methods and those that are used in practice. However, it is important to highlight that all of these methods are essentially different in that they are designed to achieve different goals (power, balance, or ethics). In all simulations, we use a uniform Beta(1,1) prior for each arm and we compute the allocation probabilities for CARA FLGI using a Monte-Carlo approximation based on 10^2^ replications. For the CARA procedures in (2)–(5), the first 50 patients are randomized following an ER procedure. After those 50 allocations, the maximum likelihood estimators of *α_t_* and *β_t_* are computed, and the associated estimate of the treatments success rates *p_t_*(*z*) is sequentially used for computing the allocation probabilities.

### 4.1 The Sepsicool Trial

The Sepsicool trial, as reported by Schortgen et al. (2012), evaluated the effect of fever control by external cooling on vasopressor requirements in septic shock. Septic shock, defined as sepsis with cardiovascular failure requiring vasopressor infusion, has a high mortality rate (40–60%). Current recommendations focus on the first few hours of sepsis management but the criteria for vasopressor selection remains debated. The original trial allocated 200 patients between the two arms with a fixed and equal randomization probability of 1/2. The primary endpoint (number of patients with 50% decrease in the baseline vasopressor dose) was available 48 hours after randomization. The secondary endpoints included all-cause mortality on day 14. At the end of the trial, the difference in the primary endpoint between treatments was not found to be statistically significant. However, day 14 mortality rate was significantly lower in the cooling group (19% vs. 34%). Furthermore, post hoc analyses adjusting on the baseline vasopressor dose differed significantly between treatment groups, indicating that the significant effect of cooling was more pronounced in patients having the lowest baseline vasopressor doses (i.e., those that had a lower illness severity). The reported odds-ratio (OR) before and after adjustment for covariates with baseline imbalances (a combination of dose and illness severity), respectively, were 0.44 and 0.36.

We show the impact of redesigning the Sepsicool study to account for such covariate data using the CARA GI and CARA FLGI and the alternative CARA patient allocation rules. We dichotomize patients by dose-severity into two groups: high or moderate versus low illness severity-dose combination. We let covariate *z* take the value 0 when a patient is from the high or moderate severity group. We report the results for two scenarios of parameter values: *α_t_* = 0.6482, *β_t_* = 0 for *t* = 0, 1 (Scenario 1) and *α*_0_ = 0.6482, *β*_0_ = 0, *α*_1_ = 1.6702, *β*_1_ = −0.3793 (Scenario 2). Scenario (1) represents the null hypothesis where there is no treatment effect nor treatment–covariate interaction while Scenario 2 represents a possible parameter realization compatible with the values reported after the Scepsicool trial. The values for Scenario 2 are arbitrary but chosen so as to be consistent with the overall and adjusted ORs reported by the trial in Schortgen et al. (2012), as response data per covariate group is not publicly available, and neither is the information on the covariate distribution. Therefore we assume that patients are equally distributed between these two groups (i.e., *p_z_* = 0.5).

The sample size was chosen to be of *N* = 450 so that ER achieves at least 90% in Scenario 1. For the CARA FLGI, we fix the block size to *b* = 10, 30, 45, 50 patients and the number of interim analysis to *J* = 45, 15, 10, 9. For SPBD the block size was set to *m* = 10.

Hypothesis testing was performed using a normal cut-off value (when appropriate) and using an adjusted version of Fisher’s exact test for comparing two binomial distributions. Fisher’s test has an actual rejection rate far below the nominal significance level. To make the designs comparable and suitable for response-adaptive bandit rules we chose its cutoff value from simulations so as to achieve the nominal type I error rate (Villar et al., 2015). Under the null, p* is defined as the mean proportion of patients assigned to the control group, whereas under the alternative, p* is computed as the mean proportion assigned to arm 1.

### 4.2 Results

Table 1 displays the results from 5000 replications of the trial. As expected, under Scenario 1 (bottom of Table 1) all the designs are equal in terms of ENF and p* (ENF is *T* × 0.3434 = 154.53). All rules allocate on average the same proportion of patients with a given covariate value to each treatment (close to 0.50). The CARA Gittins index designs have a variability in the allocation probabilities between 5 and 10 times larger than the other CARA designs.

The most interesting differences among these designs occur under Scenario 2 (top of the table). We find that the CARA GI and CARA FLGI procedures decrease the expected number of deaths dramatically compared to ER (approximately 30 less deaths on average).

The efficiency of procedures under Scenario 2 was measured by the average power of the test to reject the null hypothesis of no covariate-adjusted treatment difference (i.e., *α*_1_ – *α*_0_ = 0). Of particular interest is the potential conflicts among balance, efficiency, and ethics goals that result from the different methods. All CARA designs are more variable than the rules that achieve balance. Some of them achieve slightly higher levels of power and similar values of ENF than ER (CARA 3) while others offer a slightly reduced power accompanied by a reduction in their ENF (CARA 1) when compared to ER. As expected, the designs that have the smallest ENF (and therefore the highest performance in terms of the ethical goal) and p* are the Gittins index-based ones. However, these rules also have the largest variability of allocation probabilities and the lowest power levels. Naturally, these designs also have a much larger variability and lower power than CARA procedures designed to optimize patient benefit while simultaneously achieving a power constraint (as CARA 4 does). However, this larger variability is associated with highly skewed patient allocations only under *H*_1_.

These results suggest that the conflict between power and balance can be less acute than that between balance/power and ethics, but just as in the two-armed case with no covariates, a conflict between both of these objectives and the ethical one will always be present. In other words, in a two-armed context any modification of the CARA GI rules aimed at increasing its statistical power result in a worsening of its performance in terms of patient benefit. This power-ethics trade-off is particularly acute when the disease is fatal and rare. The CM measure suggests that only GI-based designs and TS have some trial realizations that meet this dual power-ethics criterion. Next, we explore the multi-armed case, the main motivation for this article.

### 4.3 Multi-Armed Trials: The Controlled CARA FLGI

We next create a simulation scenario to evaluate the effect of increasing the number of treatments on the ethics-power relation of the proposed CARA GI allocation methods. We consider a trial of size *N* = 300 with three arms (i.e., *K* = 2). The assumed parameters per arm are: (*α*_*t*_, *β*_*t*_) = {(–0.052, –0.473); (–1.252, 1.152); (–0.652, 1.552)} which corresponds to the following success rates vectors (0.2224; 0.3425; 0.4870) (for covariate negative patients) and (0.4750; 0.7109; 0.3717) (for covariate positive patients). Therefore, in this scenario there exists a treatment–covariate interaction: the best arm for covariate negative patients is experimental arm 2 while for covariate positive patients is experimental arm 1. As in the previous section, we assume that patients are equally distributed between these two groups (i.e., *q* = 0.5).

We compare the CARA FLGI procedures to ER, SPBD, and TS. Additionally, we heuristically extend CARA 1 and CARA 2 to, respectively, use the following allocation targets:

(9) $$\rho_{t}^{1}(z)=\frac{p_{t}(z)/q_{t}(z)}{\sum_{t=0}^{K}p_{t}(z)/q_{t}(z)},\;\rho_{t}^{2}(z)=\frac{\sqrt{p_{t}(z)}}{\sum_{t=0}^{K}\sqrt{p_{t}(z)}}.$$

Notice that these heuristics are no longer expected to optimal, as shown in Tymofyeyev et al. (2007) for multi-arm case with no covariate variables the extension is far from trivial. Procedures CARA 3 and CARA 4 are not simply extended into a heuristics rule for a multi-armed context and therefore not included in this section. In addition to the CARA FLGI rule defined by (4), we shall consider a controlled group allocation rule (CARA CFLGI) which, similarly to the CFLGI introduced in Villar et al. (2015), protects the allocation to the control treatment so that it remains fixed at $\frac{1}{\left(K+1\right)}$ per covariate group during the trial.

Table 2 reports the results of 5000 replications of this trial under the scenario above described and for each of the CARA designs considered. The operating characteristics reported are the same as before with the exception of the efficiency and ethics measures. The efficiency is measured in this case by the average marginal statistical power of the test used to detect that arm 2 is best for covariate negative patients (1 – *β*_0_) or to detect that arm 1 is best for covariate positive patients (1 – *β*_1_). The ethical performance is measured by the expected number of successes (ENS) rather than ENF. CM counts realizations that: (1) assigned 75% or more covariate 0 patients to their best arm and (2) had a statistically significant result.

As expected, the CARA GI-based rules perform extremely well in terms of patient benefit (with CARA GI achieving on average 38 more successes than a ER design) and extremely poorly in terms of marginal power and the variability of the resulting allocations. On the other hand, CARA 1 and CARA 2 have lower and similar levels of variability yet they result in different performances in terms of patient benefit and marginal power. The controlled class of CARA FLGI allocation rules results in higher values of marginal power than ER for both patient subgroups while offering and advantage in terms of patient benefit of 13–16 more successes on average depending of the block size selected. Notice that these controlled procedures achieve variability levels similar to CARA 1 and CARA 2.

## 5 Extensions: Polychotomous Covariates and Multiple Covariates

Let *Z_n_* ~ *Multinomial*(*q*_0_, *q*_1_, … , *q_M_*) for *Z_n_* ∈ {0, 1, … , *M*} where *M* is a finite number, that is, *P*(*Z_n_* = *i*) = *q_i_* for *i* = 0, 1, … , *M*. A CARA GI heuristic solution as in Section 2 for this case can be defined by considering that for every treatment–covariate combination there exists a independent arm, indexed by its covariate value and treatment arm label, that is, *zt.* Therefore, there will be (*M* + 1)(*T* + 1) arms in this reformulated the classic multi-armed bandit problem, each of which has a success rate *p_zt_*.

If there are *C* possible covariate variables and **Z_n_** is a *C* × 1 vector, where *C* is a finite number. Suppose each individual covariate *c* can take *M_c_* different values where *M_c_* is finite and specific for each covariate. A CARA GI heuristic solution as in Section 2 for this case can be defined by redefining all the covariates into a single covariate having *S* = *M*_1_ × *M*_2_ × … × *M_C_* stratification levels. Again, we consider that for every treatment–covariate level there exists a independent arm, indexed by its covariate value and treatment arm label. Therefore, there are *S*(*T* + 1) arms in this reformulated multi-armed bandit problem.

In both cases, the GI CARA procedure assigns patient *n* with covariate *z_n_* to the arm available given its covariate profile with the highest Gittins index after observing patient’s *n* − 1 outcome. The computationally feasibility of the Gittins index rule ensures the tractability of the CARA GI procedure regardless of the size of *M* or *M_c_*.

## 6 Discussion

Over the past few years, “*precision medicine*” (the tailoring of medical treatment to the individual characteristics of each patient) has shown promise through the approval of new cancer therapies for patients with specific genetic mutations. The challenge to precision medicine is that many promising new treatments have relatively few patients to test them, and even fewer patients when a treatment works only within a biomarker subgroup. Novel methodology for trial design that is able to identify superior treatments more quickly, mainly treatments that work better within subgroups, is an essential requirement to make precision medicine possible.

Non-myopic block adaptive randomization procedures based on the Gittins index have recently been proposed to offer patient benefit within a trial by more quickly identifying a superior arm if it exists. In this article, we introduce a bandit-based CARA design which can incorporate biomaker information that is potentially predictive of patient outcome. Through simulations, we illustrate how the proposed CARA FLGI procedure enables adaptively block-randomized clinical trials that are statistically conservative when no superior treatment exists and highly ethical in terms of patient benefit. Such a procedure satisfies the dual goals of differential treatment responses while satisfying an ethical imperative.

A limitation of the resulting CARA design is its high variability leading to a corresponding power loss. Although in the two-armed context this reduced power is unavoidable without a corresponding sacrifice in patient benefit, in a multi-armed context, these CARA designs offer important simultaneous patient benefit and power gains over a traditional design. A caveat to these results is that they require a temporal homogeneity of the blocks of patients recruited, as a time trend could cause an inflation of the type I error.

The derivation of the allocation probabilities with the proposed CARA design is considerably more complex than that of the existing alternative procedures. However, its practical implementation is as feasible as that of other methods, given its low computational cost. Moreover, the patient benefit advantages of the proposed design grow with the number of arms under study. In cases in which the endpoint is survival and there are very few patients with the condition, designs that prioritize patient benefit and allocate more patients to the best treatment according to their covariate profile, as the proposed CARA FLGI does, should be carefully considered as part of trial design.

We have demonstrated that the CARA FLGI can have favorable effects in a redesigned clinical trial, with minimal computational difficulties. The careful biostatistician can consider the relative benefits of such a design; if power is the most important component, then the methods of Zhang et al. (2007) present one alternative CARA method.

## Supplementary Material

Example code and instructions for running it are available with this article as a web supplement at the *Biometrics* website on Wiley Online Library.

Supplementary information

## Figures and Tables

**Table 1:** Redesigning the Sepsicool trial. Results from 5000 replications for different CARA procedures, *N* = 450

Alternative								

Procedure	*b*	(1 − *β*)	*N*_0_(*N*)/*N* (s.d)	*N*_0t*_ (*N*)/*N* (s.d)	*N*_1t*_ (*N*)/*N* (s.d)	ENF (s.d)	p* (*s.d*)	CM

ER	1	0.91	0.500 (0.02)	0.500 (0.03)	0.500 (0.04)	119.22 (9.24)	0.500 (0.02)	0.0
SPBD	10	0.90	0.500 (0.02)	0.500 (0.02)	0.500 (0.02)	119.14 (9.39)	0.500 (0.02)	0.0
CARA 1	10	0.88	0.330 (0.06)	0.697 (0.09)	0.626 (0.09)	106.94 (10.23)	0.662 (0.07)	1.5
CARA 2	10	0.91	0.476 (0.03)	0.528 (0.04)	0.520 (0.04)	117.54 (9.36)	0.524 (0.03)	0.0
CARA 3	10	0.91	0.453 (0.03)	0.560 (0.04)	0.532 (0.04)	115.81 (9.50)	0.547 (0.03)	0.0
CARA 4	10	0.89	0.391 (0.04)	0.638 (0.06)	0.578 (0.06)	111.02 (9.91)	0.609 (0.04)	0.0
TS	10	0.89	0.301 (0.06)	0.737 (0.08)	0.660 (0.09)	104.68 (9.42)	0.699 (0.06)	6.2
CARA FLGI	50	0.44	0.142 (0.09)	0.810 (0.19)	0.904 (0.07)	92.30 (9.53)	0.858 (0.09)	38.0
CARA FLGI	45	0.38	0.123 (0.10)	0.896 (0.15)	0.856 (0.16)	91.90 (10.71)	0.877 (0.10)	28.0
CARA FLGI	30	0.42	0.108 (0.09)	0.851 (0.19)	0.931 (0.05)	90.30 (10.13)	0.892 (0.09)	30.0
CARA FLGI	10	0.20	0.133 (0.17)	0.902 (0.23)	0.830 (0.27)	90.94 (13.96)	0.867 (0.17)	18.0
CARA GI	1	0.18	0.102 (0.14)	0.930 (0.16)	0.864 (0.25)	91.54 (12.84)	0.898 (0.14)	14.0

Null								

Procedure	*b*	*α*	*N*_0_(*N*)/*N* (s.d)	*N*_0t*_ (*N*)/*N* (s.d)	*N*_1t*_ (*N*)/*N* (s.d)	ENF (s.d)	p* (*s.d*)	CM

ER	1	0.05	0.500 (0.02)	0.500 (0.03)	0.500 (0.03)	154.33 (9.95)	0.500 (0.02)	0.0
SPBD	10	0.05	0.500 (0.02)	0.501 (0.02)	0.500 (0.02)	154.58 (9.95)	0.500 (0.02)	0.0
CARA 1	10	0.05	0.500 (0.06)	0.500 (0.09)	0.500 (0.09)	154.44 (10.22)	0.500 (0.06)	0.0
CARA 2	10	0.05	0.500 (0.03)	0.500 (0.04)	0.500 (0.04)	154.28 (10.03)	0.500 (0.03)	0.0
CARA 3	10	0.05	0.500 (0.02)	0.500 (0.03)	0.499 (0.04)	154.53 (10.13)	0.499 (0.03)	0.0
CARA 4	10	0.05	0.500 (0.04)	0.500 (0.05)	0.499 (0.05)	154.41 (10.05)	0.499 (0.04)	0.0
TS	10	0.06	0.501 (0.07)	0.501 (0.10)	0.501 (0.10)	154.61 (10.14)	0.501 (0.07)	0.0
CARA FLGI	50	0.06	0.555 (0.28)	0.523 (0.24)	0.490 (0.33)	155.76 (11.23)	0.522 (0.24)	4.0
CARA FLGI	45	0.04	0.533 (0.23)	0.522 (0.34)	0.544 (0.29)	155.42 (10.22)	0.533 (0.23)	4.0
CARA FLGI	30	0.04	0.481 (0.35)	0.535 (0.27)	0.586 (0.38)	154.22 (10.10)	0.535 (0.27)	2.0
CARA FLGI	10	0.04	0.467 (0.31)	0.463 (0.42)	0.472 (0.39)	152.52 (9.44)	0.467 (0.31)	4.0
CARA GI	1	0.06	0.512 (0.29)	0.555 (0.40)	0.467 (0.42)	154.78 (10.28)	0.512 (0.29)	2.0

**Table 2:** Results from 5000 replications for different CARA procedures, *N* = 300: arm 1 is best for covariate positive patients and arm 2 is best for covariate negative patients

Procedure	*b*	(1 − *β*_0_)	(1 − *β*_1_)	*N*_0_(*N*)/*N* (s.d)	*N*_0t*_ (*N*)/*N* (s.d)	*N*_1t*_ (*N*)/*N* (s.d)	ENS (s.d)	p*(*s.d*)	CM
ER	1	0.77	0.69	0.334 (0.03)	0.334 (0.04)	0.333 (0.04)	132.01 (8.44)	0.334 (0.03)	0.0
SPBD	12	0.75	0.71	0.334 (0.02)	0.333 (0.03)	0.333 (0.03)	131.91 (8.36)	0.333 (0.02)	0.0
CARA 1	10	0.64	0.74	0.221 (0.06)	0.507 (0.11)	0.566 (0.09)	149.15 (9.64)	0.538 (0.07)	0.2
CARA 2	10	0.67	0.73	0.296 (0.05)	0.398 (0.07)	0.388 (0.05)	137.39 (8.62)	0.393 (0.04)	0.0
TS	10	0.71	0.75	0.224 (0.05)	0.485 (0.09)	0.592 (0.09)	149.28 (9.49)	0.542 (0.06)	1.0
CARA CFLGI	50	0.78	0.74	0.340 (0.02)	0.513 (0.11)	0.597 (0.05)	149.98 (8.27)	0.558 (0.06)	0.0
CARA CFLGI	10	0.82	0.88	0.331 (0.03)	0.516 (0.13)	0.637 (0.05)	151.44 (9.43)	0.580 (0.06)	0.0
CARA FLGI	50	0.32	0.02	0.100 (0.04)	0.653 (0.20)	0.843 (0.06)	167.06 (10.72)	0.754 (0.09)	5.0
CARA FLGI	30	0.30	0.04	0.085 (0.05)	0.688 (0.25)	0.878 (0.08)	167.86 (9.46)	0.789 (0.12)	8.0
CARA FLGI	20	0.16	0.04	0.100 (0.07)	0.711 (0.23)	0.866 (0.09)	168.40 (9.51)	0.793 (0.11)	4.0
CARA FLGI	10	0.24	0.02	0.068 (0.05)	0.776 (0.18)	0.885 (0.13)	171.72 (11.25)	0.834 (0.11)	4.0
CARA GI	1	0.08	0.04	0.054 (0.04)	0.704 (0.29)	0.937 (0.06)	171.42 (11.80)	0.828 (0.14)	2.0
